# Using Liver Organoids as Models to Study the Pathobiology of Rare Liver Diseases

**DOI:** 10.3390/biomedicines12020446

**Published:** 2024-02-17

**Authors:** Dalia A. Obeid, Tanveer Ahmad Mir, Alaa Alzhrani, Abdullah Altuhami, Talal Shamma, Sana Ahmed, Shadab Kazmi, Iriya Fujitsuka, Mohd Ikhlaq, Mohammad Shabab, Abdullah M. Assiri, Dieter C. Broering

**Affiliations:** 1 Tissue/Organ Bioengineering and BioMEMS Lab, Organ Transplant Centre of Excellence, Transplant Research and Innovation Department, King Faisal Specialist Hospital and Research Centre, Riyadh 11211, Saudi Arabiaabaltuhami@kfshrc.edu.sa (A.A.); sana.ahmed32@gmail.com (S.A.);; 2 College of Medicine, Alfaisal University, Riyadh 11533, Saudi Arabia; 3 College of Applied Medical Sciences, King Abdulaziz University, Jeddah 21423, Saudi Arabia; 4 School of Materials Science, Japan Advanced Institute of Science and Technology, Nomi 923-1292, Ishikawa, Japan; 5 Department of Child Health, School of Medicine, University of Missouri, Columbia, MO 65212, USA; 6 Toyama Red Cross Hospital, Toyama 930-0859, Toyama, Japan; 7 Graduate School of Innovative Life Science, University of Toyama, Toyama 930-8555, Toyama, Japan; 8 School of Pharmacy, Desh Bhagat University, Mandi Gobindgarh 147301, Punjab, India

**Keywords:** organoids, liver, rare disease modeling, biomaterials, regenerative medicine

## Abstract

Liver organoids take advantage of several important features of pluripotent stem cells that self-assemble in a three-dimensional culture matrix and reproduce many aspects of the complex organization found within their native tissue or organ counterparts. Compared to other 2D or 3D in vitro models, organoids are widely believed to be genetically stable or docile structures that can be programmed to virtually recapitulate certain biological, physiological, or pathophysiological features of original tissues or organs in vitro. Therefore, organoids can be exploited as effective substitutes or miniaturized models for the study of the developmental mechanisms of rare liver diseases, drug discovery, the accurate evaluation of personalized drug responses, and regenerative medicine applications. However, the bioengineering of organoids currently faces many groundbreaking challenges, including a need for a reasonable tissue size, structured organization, vascularization, functional maturity, and reproducibility. In this review, we outlined basic methodologies and supplements to establish organoids and summarized recent technological advances for experimental liver biology. Finally, we discussed the therapeutic applications and current limitations.

## 1. Introduction

The liver is the largest gland that controls a multitude of critical biological functions in the human body, including the metabolism, storage of essential nutrients, regulation of blood volume and immune responses, lipid and cholesterol hemostasis, and biotransformation [1]. It is well documented that the mammalian liver possesses a powerful regenerative potential in terms of the recovery of mass and function using a variety of modes of regeneration depending on the size of resection, the degree of injury, and the extent of organ compromise [2]. However, the regenerative capacity of the liver can be severely impaired by several factors, including infection, toxic insult, immune system malfunction, genetic disorders, and tumorigenesis, which can result in irreversible damage [3]. Thus, liver-associated diseases cause approximately 2 million deaths yearly [4]. The allogeneic liver transplantation strategy is widely considered the only restorative treatment option available for eligible patients with severe complications related to chronic and end-stage liver diseases. In fact, the liver is the second most frequently transplanted organ after the kidneys, with almost 34,694 yearly procedures conducted globally [5]. However, the availability of medically suitable donated organs or the challenge of preventing graft rejection limit this life-saving treatment [6,7,8].

Structurally, the liver is a complex organ that primarily comprises ~80% parenchymal (hepatocytes) and 6.4% nonparenchymal cells, including Kupffer cells, liver endothelial cells, and hepatic stellate cells. Moreover, 40% of liver cells are localized in the tissue’s sinusoidal compartment, while the hepatic sinusoid walls are lined by nonparenchymal cells [9]. Parenchymal cells play key roles in major hepatic functions, while nonparenchymal cells coordinate with hepatocytes and support parenchymal cells in maintaining the liver structure and physiology. Both parenchymal and nonparenchymal cell types interact with one another to establish a controlled microenvironment that efficiently regulates cellular activities throughout the entire organ [10]. Hepatocytes are considered the most important cell type in parenchymal tissues as they perform most liver functions [9,10]. On the other hand, cholangiocytes are epithelial cells that form the lining of the bile ducts. Both cholangiocytes and hepatic stellate cells (HSCs) play key roles in liver regeneration during chronic liver injury. HSCs are stimulated by inflammation and can produce hepatocyte growth factor (HGF) in response to severe injury. At the same time, cholangiocytes are converted into hepatocytes through a bi-phenotypic state to aid in regeneration [11]. The damage or destruction of the parenchymal and nonparenchymal cells because of several abnormalities necessitates the development of long-term culture systems to study liver biology and pathophysiology [11].

Over the past two decades, medical research has advanced in many areas using cell lines based on 2D monolayer cultures and animal models [12]. Adopting cell-line-based experimental strategies has greatly aided in the understanding of cellular pathways, especially in cancer research. Cell lines are generally less costly and more readily available than animal models [13]. A common way to study a disease is to start with cell lines and advance into animal models. However, cell lines lack complexity, and complicated biological mechanisms cannot be fully understood using two-dimensional cell culture models. Moreover, animal models have contributed significantly to medical research and development but cannot fully mimic human disease pathogenesis [14]. Challenges in using animal models also include ethical considerations, cost, availability, knowledge of the model, and reproducibility. Development in gene editing allowed for both cell lines and animal models to thrive and become humanized models [15,16]. Although there have been recent advances in understanding liver biology and developing different basic and preclinical research approaches to study the normal physiology and pathology of the liver, the anatomical complexity and genetic variabilities in humans make investigating rare diseases a tough challenge. Thus, most human-specific rare genetic disorders have limited therapeutic options and a poor prognosis [17]. Hence, bioengineering complex and miniaturized liver models with adequate performance compliance requirements has become crucial to minimize the current liver-transplantation-based treatment challenges. Numerous tissue engineering and regenerative medicine technologies are being actively explored to address the issues associated with conventional 2D or animal models [18,19,20,21,22].

The emergence of bioengineered organoid models using patient-specific samples offers great potential to generate personalized models [14,23,24,25]. Several researchers are intensively working with 3D organoid cultures for various biomedical applications, including disease modeling, biopharmaceuticals, liver repair, or regeneration [15,26]. In this concise review, we provided an overview of organoid culture systems for liver research. We then summarized the essential biocomponents and methodologies for generating liver organoids. Furthermore, we discussed organoid technology’s recent advances, advantages, and challenges.

## 2. Overview of Organoid Culture System

Organoids refer to an in vitro three-dimensional cell culture system derived from adult stem cells, embryonic stem cells, or induced pluripotent stem cells that are capable of self-renewal and self-organization and recapitulate the genotypic and phenotypic characteristics of the native tissue or organ [14,27]. The advent of organoid technology is one of the most significant scientific advances with powerful potential to revolutionize research fields of experimental biology, tissue engineering, and translational regenerative medicine [14]. Huch’s team used mouse livers to establish the first generation of liver organoids derived from adult stem cells (Lgr5+) [28]. The research group expanded genetically stable organoids in an R-Spondin-1-based conditioned culture medium [28]. The second breakthrough in the field was the generation of liver organoids from iPSCs, reported by Takebe’s team in the same year [29]. Since then, several research groups have developed different methods to culture liver organoids, allowing them to become novel tools for personalized applications [27,28,29,30].

The general procedure for producing organoids in culture is as follows: (1) the selection of a cell source and culture system, (2) the addition of precise soluble factors, (3) induction, (4) proliferation, and (5) expansion [30]. Organoids’ functionality depends on the generation of mature cells, the formation of liver-specific cell markers, and structural organization. Liver organoids with mature profiles can produce liver enzymes such as albumin and ALT/AST [30]. In addition, genetic markers can be evaluated at the RNA level. For example, cholangiocytes express the KRT19, KRT7, and SOX9 genes, while hepatocytes express the ALB, HNF4A, and MRP4 genes [31]. Finally, the maturity of the structural organization of the organoids can be confirmed via magnification, and depending on the culture system, the morphology of the organoids should be spherical and surrounded by endothelial cells (ECs) or vasculature [32].

### 2.1. Essential Components and Methodologies for Growing Organoids

#### 2.1.1. Cell Source

Stem cells are a population that can differentiate into all specialized cell types in culture conditions. These cells are generally characterized by their inherent self-renewal and self-organization capabilities [33]. These unique features of stem cells are fundamental for their adoption in organoid research for various biomedical applications. There are two main types of pluripotent stem cells (PSCs): (i) embryonic stem cells (ESCs) (derived from the inner cell mass of animal embryos) and (ii) induced pluripotent stem cells (iPSCs) (generated through the reprogramming of mouse and human somatic cells). Although, both ESCs and iPSCs can be employed to produce liver organoids. However, technical and ethical issues hamper the widespread use of ESCs. On the other hand, iPSCs are generated from somatic cells, and the ectopic expression of critical reprogramming factors (*OCT4*, *SOX2*, *KLF4*, and c-*MYC*, or *OCT4*, *SOX2*, *NANOG*, and *LIN28*) overcome the limitation associated with ESCs [34,35,36]. In an interesting study, researchers compared cell sources such as ESCs, adult stem cells (ASCs), and iPSCs to assess the suitability of their novel culture systems for liver research. The authors found that ESCs and iPSCs preserved liver growth and functions. However, both cell types showed fewer liver-specific functions than ASC-derived organoids [31].

**Figure 1:**
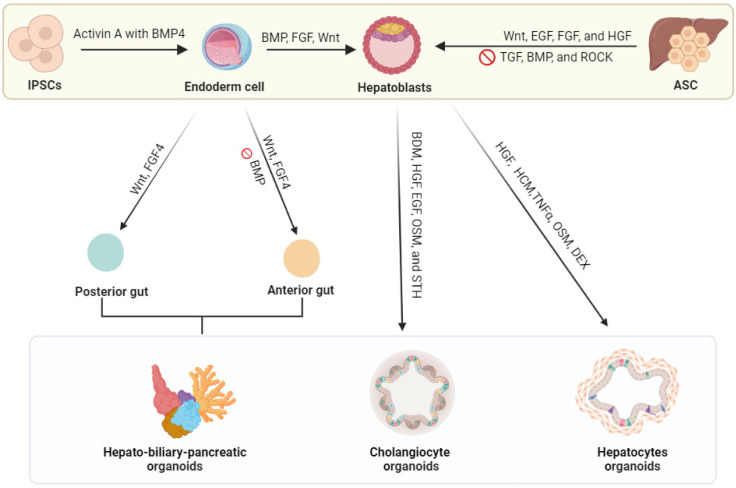
Schematic diagram of in vitro liver organoid model creation. Various cell types (induced by pluripotent cells, hepatocytes, cholangiocytes, and adult stem cells or Lgr5+ liver stem cells) are the primary cell sources commonly used to generate organoids. Excised or biopsy specimens from humans (healthy or diseased patients) are used to obtain cells for organoid growth by incubating them with various signaling factors. Abbreviations: FGF, fibroblast growth factor; BMP, bone morphogenic protein; Wnt, noncanonical Wnt/calcium pathway; VEGF, vascular endothelial growth factor; HGF, hepatocyte growth factor; FGF, fibroblast growth factor; TGF, transforming growth factor; ROCK, ROCK signaling pathway regulates cell morphology; BDM, 2,3-butanedione monoxime; OSM, oncostatin M; STH, somatotropin; HCM, hepatocyte culture medium; TNF-α, tumor necrosis factor alpha; DEX, dexamethasone.

Therefore, ASCs or iPSCs are considered powerful cell sources to generate liver organoids for patient-specific disease modeling, bioassays, and pharmaceutical studies (Figure 1) [15,37,38,39,40,41,42]. In liver organoid research, organ-specific ASC progenitors, especially bile-duct-cell-derived progenitors (Lgr5+ cells), are commonly employed for organoids’ growth and long-term expansion. Due to their high stability, ASC-derived liver organoids can be differentiated into functional hepatocytes for both in vivo and in vitro modeling. It has been demonstrated that liver organoids derived from ASCs retain genomic stability at different stages [43]. In general, the use of ASCs is currently the most common because they can be directly obtained from a patient source, thus reducing the cost of cell editing or gene manipulations [44].

In addition, immortal liver cell lines can also generate liver organoids. Immortal cells are epithelial cell lines derived from tumorous cells that do not stop growing and dividing or are artificially manipulated to proliferate indefinitely and can thus be cultured for several generations. [45]. The most common liver cell lines are HepG2 and Huh7; these cells are cancerous and primarily used in 2D (monolayer) culture systems. Organoids for hepatocellular carcinoma (HCC) models can be established from HCC cell lines, such as Hep3B, Huh7, and HepG2 [46]. The ability to use these cell lines for organoids will significantly reduce the cost as they need fewer growth factors compared to iPSCs and ASCs. However, immortalized cell lines have many limitations, including genetic instability with time [46]. Developing cell-line-based organoids could be much easier and cheaper [47]. Although immortalized cell lines are limited in their ability to model some diseases, they may be useful for cancer research because of their important features in human cancer and development [48].

#### 2.1.2. Soluble Factors

In organoid technology, specific soluble growth factors (GFs) are critical in formulating organoid culture media. In eukaryotic organisms, growth factors are primarily secreted by the host as a culture medium. This medium possesses different amounts of specific bioactive components for each cell type. Researchers employ stepwise differentiation protocols to produce liver organoids by supplementing the culture medium with particular growth factors. The choice of growth factors depends on existing scientific knowledge of the role of each growth factor in molecular pathways that control embryonic development and growth. For example, physiological stress can induce the angiogenesis and secretion of regeneration-associated factors in the liver, leading primary hepatocytes to enter the life cycle [49]. The critical pathways that promote the growth, proliferation, viability, maintenance, migration, and maturation of hepatic progenitors in the liver are directly linked to the functional roles of FGF, HGF, Wnt, BMP, RA, and TGFβ signaling [49]. Supplementing the organoid medium with the mitogenic EGF growth factor is critical for growth and proliferation in vitro [39]. Conversely, blocking the Notch signaling pathway for human cell differentiation helps to polarize hepatic progenitor cells to a hepatocyte phenotypic fate [39].

Moreover, the growth factors in organoid cultures depend on the cell sources, such as iPSCs or ASCs. Organoids derived from iPSCs must differentiate into germ layer specification (endoderm, mesoderm, or ectoderm), followed by maturation [48]. After germ specification, growth and signaling factors are generally added into the media to generate specific cell types. For iPSC germ layer specification, Activin A can be added to the endoderm layer, Activin A with BMP4 can be added to the mesoderm layer, and Wnt with BMP4 can be added to the ectoderm layer [48]. For liver organoids (derived from the endoderm), Activin A and BMP4 should be added [48]. The second step is to add tissue-specific growth factors to activate particular signaling pathways, such as Wnt, BMP, and FGF [48]. Endothelial cells are another excellent example of cells that can be driven from iPSCs. Endothelial cells can be generated from the differentiation of mesoderm cells into angioblasts by adding FGF2, BMP4, Activin A, and VEGF [50]. Angioblasts can then be differentiated into endothelial cells by growing the cells in a medium that is rich in VEGF factors and TGFβ inhibitors [50].

For ASC-driven organoids, tissue-specific growth factors are either induced or inhibited. The upregulations of the Wnt, EGF, FGF, and HGF pathways for human liver organoid development are commonly supplied as signaling components for cell differentiation and niche functions [13,32,43,51,52,53]. Recent studies revealed that the R-spondin-1 and BMP growth factors play pivotal roles in embryogenesis, adult tissue homeostasis, and disease [53,54]. Therefore, R-spondin-1 and BMP are widely used as essential components of organoid culture media [53]. R-spondin-1 regulates the Wnt pathway activity in epithelial stem cells, and BMP repressors such as Noggin or Gremlin 1 inhibit differentiation cues from the BMP pathway [54]. The summary of the differentiation scheme for either cell types into hepatocyte-like cells, cholangiocyte-like cells, or a complex structure is shown in Figure 1.

Other liver resident cells, like Kupffer cells, can also be generated from iPSCs with specific growth factor cocktails [13]. The first step is the addition of BMP-4, VEGF, SCF, and ROCK inhibitors to develop embryoid bodies. The second step is the addition of M-CSF, IL-3, and β-mercapto-ethanol to create pre-macrophage growth factors to enhance growth. Lastly, the pre-macrophages should be cultured in a mix of primary human hepatocyte media and advanced DMEM media to generate induced Kupffer cells [13].

Although cells can be derived from the same source, adding different growth factors in the media can result in cells with distinct functions and fates. In one study, two organoid cultures were derived from the same human liver progenitor cells [52]. One population was overstimulated with Wnt medium and became extrahepatic cholangiocyte organoids (ECOs), while the other grew in ordinary media and became intrahepatic cholangiocyte organoids (ICOs). Both organoids had cholangiocyte fate differentiation capacity, but ECOs lacked the potential for differentiation towards a hepatocyte-like fate. This example shows how even a little change in the medium can result in a diverse organoid population that can be used for many applications. It is challenging to isolate and propagate functional primary cholangiocytes alone for the long term. Nevertheless, one group was able to differentiate human iPSCs into cholangiocyte fate by using biliary specification growth factors like Activin A, retinoic acid, and FGF-10. These factors successfully activated the signaling pathways of hepatocytes and hepatic cellular markers [53].

### 2.2. Biomaterials as Substrates for Growing Organoids

The current gold standard methods of embedding liver organoid models rely excessively on Matrigel as a 3D matrix for microenvironmental modulation [55]. Matrigel is considered a magic or highly bioactive material that supports organoids’ growth, expansion, or proliferation and is derived from different sources. However, organoids cultured in Matrigel are unsuitable for downstream clinical applications due to the dependence on the murine tumor origin of the Matrigel matrix [56,56]. Another major challenge of using non-specific ECM (Matrigel) is the need for more control over the composition and stiffness of the gel. Developing high-performance biomaterials for organoid research is significant for the high-end manufacturing of organoid-based bioengineered products [55,56]. Several investigators are working on developing alternative biomaterials using natural biopolymers or synthetic or semisynthetic polymers [57,58,59,60]. Naturally sourced biomaterials can be created using an organ-derived extracellular matrix, collagen, alginate, collagen–laminin–fibroin, fibrin–laminin, and hyaluronan [55]. Although most natural-source-derived biomaterials exhibit excellent bioactive properties and offer higher biocompatibility performance, pure biopolymers exhibit several shortcomings, including poor stability, mechanical integrity, and unmanageable degradation [55,56].

For liver organoid research, a combination of natural and synthetic polymers are suitable substitutes for Matrigel. Synthetic polymers exhibit high structural or mechanical integrity and processability [57,58]. Using synthetic polymers for organoid culture might be the most valuable method from the perspective of availability and reproducibility. The most common synthetic polymers used to create organoid culture microenvironments include poly-ethylene-glycol (PEG), poly-vinyl-alcohol (PVA), and poly-N-isopropyl acrylamide (PNIPAAM) [58]. Gjorevski and Lutolf customized enzymatically cross-linked PEG hydrogels that are compatible with cell growth [59]. The authors prepared the hydrogel with a synthetic polymer with bioinstructive signals required for iPSC expansion and organoid formation. Another interesting study demonstrated the synthesis of a tailorable hybrid hydrogel with both natural and synthetic components [60]. The authors incorporated PEG–gelatin, PEG, and lysine to establish a complex extracellular matrix-like microenvironment. The results showed that hybrid hydrogels supported organoid growth and expansion, and the data were comparable to Matrigel in terms of complexity and long-term culture. Hydrogel capsules have also been reported to make one-step composite hydrogel capsules using Na-alginate (NaA), chitosan (CS), fibrinogen, and thrombin (CHC) [61]. In this study, the iPSCs encapsulated in the microcapsules displayed high stability and biocompatibility.

The critical characteristics of the microenvironment matrix for organoid culture are determined by the target material and the methodology employed for its synthesis. The prerequisites for selecting biocompatible materials to develop culture environments for organoid research applications depend on the cell source, material composition, surface chemistry, hydrophobicity, degradation rate, and intended research. The exploration of materiobiology in the field of organoids has only just begun, and intensive research in this area is needed.

### 2.3. Bile Duct-Like Cystic Structures and Integration of Physical Cues

Many researchers have worked on improving the complexity of organoids for disease modeling by using multiple cell lineages and multi-cellular structures/buds. These multiple organoid structures can also be more complex with vascularization and by introducing cell–cell interactions. The summary of the development and complexity of organoid systems for liver research is shown in Table 1. The first layer of complexity in organoid research is the development of buds, which undergo significant vascularization and colonization by hematopoietic cells to become the primary site of fetal hematopoiesis. The earliest attempt at creating liver buds was first described by Takebe’s team in 2014 [26,62]. To make liver buds, the group successfully cultured iPSCs with hepatic cells, HUVECs cells, and MSCs. The system was working; the cells were self-organizing but lacked cellular liver functions. The establishment of vascularized and functional liver buds (LBs) in a comprehensive, scalable, and reproducible method was later achieved by the same team in 2017 using iPSCs [63]. The next level of complexity is creating a multi-cellular liver organoid, such as a hepatic–biliary–pancreatic organoid (HBPO) structure. An integrated anteroposterior model was first developed to demonstrate the generation of functional HBPOs consisting of hepatic, biliary, and pancreatic structures [64]. Although this system showed functional structures, it needed additional stromal cells, such as septum transversum mesenchyme and endothelial progenitor cells. Koike et al. (2021) continued to work on a functional HBPO by improving the model by adding endoderm and mesoderm cell populations to the structure. This adjustment helped to make the structure more refined and suitable for long-term culture [65]. The latest breakthrough in complex organoids is using multi-lineage liver organoids (mLOs) with functional vasculature and bile ducts from iPSCs [51]. This model includes hepatic endoderm cells, hepatic stellate cell-like cells (HscLCs), and endothelial cells. Furthermore, in this study, the addition of HscLCs and the regulation of Notch signaling during mLO maturation inhibited apoptotic cell death and promoted vascularization and biliary duct formation. The model developed capillary-like vessels with perfusable lumens sensitive to fibrosis-associated cytokines.

Organs and tissues in in vivo systems reside in an interacting microenvironment composed of many physical and biochemical cues with dynamic mechanical structures. Another approach is adding integrative cues to the co-culture system, and organoids can be controlled to provide a human-like microenvironment [71,72,73]. Engineering approaches to produce human-like organoid co-culture systems include adding mechanical cues, nutritional needs and metabolic cues, related tissues such as stromal and immune cells, and biosensing technology [71,72,73,74,75]. In liver research, several approaches have been used for organoid culture, including hollow fiber membranes (HF), spinning bioreactors (SBs), organ-on-a-chip (OoC), multi-organ microfluidic chips (MOCs), and microfluidic vascular beds. Table 2 provides an overview of the strategies exploited for liver organoid bioengineering for different applications.

## 3. Organoid Technology as a Tool for Modeling Rare Liver Diseases

The development of closely biomimicking rare disease models in vitro might be beneficial to effectively understand the initiation or pathogenesis of diseases and to explore options for the effective treatment of rare diseases. As mentioned above, conventional approaches for studying the pathogenesis of liver-linked disorders rely on classical cell-line-based 2D in vitro cultures or animal-based in vivo models. Traditional cell-based and animal-based disease models are limited in their applications because they lack physiological holistic components and often need to adequately reflect human tissues or organs’ histological specificity and genetic heterogeneity during normal or disease states. Organoid technology has made it possible to generate long-term culture-based laboratory models and replicate biological pathophysiological attributes of the source tissue in remarkable detail, as explored in Table 3.

Recent advances in stem cell biology have allowed researchers to harness the pluripotency of stem cells and induce them in three-dimensional culture systems (especially in Matrigel) to self-assemble into biomimetic spherical constructs or organoids [71,72,73,74,75]. Following the successful optimization of organoid culture technology, some progress has been made over the past five years in modeling liver-specific rare genetic diseases using the organoid platform [76,77,78,79,80,81,82,83,84,85,86,87,88,89]. To date, only a few laboratories have been able to generate organoids for rare disease models using original tissues or with gene editing techniques to better understand the pathogenesis of Alagille syndrome (ALGS) [76]. ALGS is a rare liver genetic disease where patients have dysfunctional bile duct formation caused by an impaired NOTCH pathway and *JAG1* mutation [76]. The model used in this study was derived from human iPSCs and was manipulated to mimic an ALGS-diseased liver. Interestingly, the authors found that the type of *JAG1* mutation itself significantly impacted the pathogenesis of liver disease [76]. Organoids with specific mutations in the *JAG1* gene showed severe liver disease and impaired development, while other organoids showed no dysfunction. The use of organoids in this study helped to recapitulate a rare genetic disorder, and also provided insight into the effects of different genetic mutations on liver function.

A similar study was carried out for modeling biliary atresia in vitro. Biliary atresia (BA) is a rare infant liver and bile duct disease [77]. As this disease occurs in infants, more models need to be developed to understand the pathogenicity and personalized drug screening. Recently, it has been demonstrated that patient-derived organoids significantly accelerated the search for potentially effective drugs [77]. Other studies helped to discover new molecular pathways involved in pathogenesis and revealed a disorder effect on epithelial development [78,79]. Another exciting and rare liver genetic disease is Wilson’s disease, which is caused by copper accumulation in the body, particularly in the liver [80,81]. The organoid model of Wilson’s disease has been reported primarily using a canine-derived source biopsy. Remarkably, the models developed for this disorder have been used mainly for experimental therapies. Furthermore, a Japanese group reported that the survival rate of the Wilson’s disease model in dogs improved after genetic manipulation and the injection of hepatocyte organoids [80]. Meanwhile, in another study, the gene supplementation of hepatic organoids in COMMD1-deficient dogs restored liver function and cured the disease [81]. Furthermore, another rare liver disease that has been well studied using the liver organoid model is Primary Sclerosing Cholangitis (PSC). PSC is a long-term liver and bile duct disease in which the liver becomes inflamed [82]. Two studies using humanized bile duct organoids allowed the researcher to determine the role of inflammation in disease development [82,83]. Furthermore, organoid models have not yet been applied to Crigler–Najjar syndrome, Galactosemia Lysosomal Acid Lipase Deficiency (LALD), glycogen storage disease, or acute hepatic porphyrias. Thus, the application of organoid models can minimize the gap in the research on these rare liver diseases.

## 4. Limitations of the Current Organoid System

Organoids are considered valuable for disease model development because they possess many advantages over existing models. For example, organoid models can be maintained in vitro for long periods, and they preserve the structural complexity and genetic characteristics of the tissues of origin. Their molecular, ultrastructural, and functional persistence may help to elucidate the developmental biology of healthy human tissues and rare disease states. Currently, the groundbreaking field of organoid research is still in its infancy. Several scientific and technological limitations hinder the clinical application of bioengineered organoids. Although published reports demonstrate that engineered organoids are equipped to execute the majority of the hepatic function, due to their micron-sized scale, there still needs to be an obvious gap between the functions of engineered organoids and their normal counterpart tissues [69,70,71,72,73,74,75,76,77,78,79,80,81,82,83,84,85,86,87,88,89]. A lack of vasculature, an imbalance in the permeation of nutrients, a lack of adequate oxygen supply to the core region, and difficulties in the excretion of metabolites are some of the other challenging issues that need to be addressed immediately [71,72,73,74,75].

Materiobiology and technological variations for organoid development are also a concern. For example, different organoid-related laboratories apply various materials and experimental techniques to generate organoids, which severely hamper the specificity and reproducibility of the engineered constructs. From a translational perspective, reproducibility is considered a fundamental requirement for clinical application. To overcome these problems, it is necessary to consider the unification of methodologies and the standardization of protocols with quality indicators. For example, various materials, growth- and differentiation-related supplements, and culture protocols are reported in scientific papers, but they are usually independently developed, and experimental strategies vary from laboratory to laboratory. Adopting a universal, animal-free culture microenvironment and adhering to standard research procedures and guidelines will help to minimize preclinical data reproducibility, replicability, and clinical validation barriers.

Another limitation is the dependence of organoid culture on 3D culture substrates. Matrigel (basement membrane matrix) is considered the gold standard 3D culture platform for organoid encapsulation. The main drawback of Matrigel is that it is extracted from Engelbreth-Holm-Swarm mouse sarcoma; the scope of Matrigel is limited to preclinical models and it is not applicable to clinical-grade production due to its origin, compositional variability, and immune-rejection-related issues. Thus, there is a growing demand to develop and validate new animal-free biomaterial products to establish clinically applicable organoids. There is a strong need for interdisciplinary collaboration among researchers from various disciplines, including stem cell biology, bioengineering, biochemical/biomaterial engineering, biomechanics, and polymer surface chemistry, to overcome this challenge.

Thus, intensive research is also needed to develop alternative extracellular mimetic biomaterials with well-defined matrix components. Finally, the cost of organoid research is another limiting factor because Matrigel, media supplements, and growth factors are very expensive. As with any new field, there are initial cost difficulties, but costs can be reduced over time and with improved protocols. Thus, developing low-cost biomaterials and small bioactive molecules offers future hope in reducing the cost of organoid research. Overall, the translational applications of organoid research are limited because of a plethora of requirements. To complement human organs’ microenvironments and cell–cell interactions, organoid models must include artificial cues. These cues can make the model more realistic for disease modeling and related studies. For example, to study immunology or infectious diseases, it is essential to include immune cells. Similarly, studying gut or skin disorders, including microflora cells, may be helpful to generate more realistic results.

## 5. Conclusions and Perspectives

In this review, we discussed the recent trends in liver organoid research and highlighted several challenging issues to motivate further developments. There is no doubt that the study of organoids is a hot research topic. Several interesting publications have comprehensively demonstrated the revolutionary potential of organoid technology in tissue development, drug toxicology, and disease modeling. Research directions towards bridging the gap between preclinical and clinical research by providing appropriate human-disease-mimicking in vitro models are fascinating and encouraging. Organoids are expected to gain more attention in basic experimental biology and applied medical research. Although organoid technology offers future hope, several shortcomings of organoid research must be resolved. To create more realistic organoid models, the following scientific and technical challenges need to be addressed: (i) cost containment by developing inexpensive bioactive molecules with growth factor-mimicking properties, (ii) the development and adoption of implantable biomaterials, (iii) the development of organoids with built-in vasculature and innervation, (iv) the development of novel strategies for the maturation organoids, (v) the co-culturing of organoids using immunocompatibility cues, and (vi) the development of novel biosensing strategies to monitor the pathology and physiology of growing organoids in real time. Addressing the above issues will open the door to broader organoid application possibilities. Fully functional organoids would also go a long way in addressing issues related to organ damage. As a result, organoids will not only be applied in preclinical research and patient-specific disease model developments or in drug screening trials, but also in organ repair and regeneration.

## Figures and Tables

**Table 1:** Summary of the progress of organoid development from single-cell to multi-cellular organoid structures.

Reference	Culture	Cell Source	Liver Organoids	Outcome
[28]	In vitro ASC expansion	ASC (Lgr5+)	Hepatocytes and cholangiocytes	Liver organoid
[62]	In vitro iPSC expansion	iPSCs, ECs, MSCs	Hepatocytes	Vascularized and functional human liver
[66]	In vitro decellularized liver matrix (LEM)	ASCs	Hepatocytes and cholangiocytes	Self-assembled liver organoids were recapitulated: hepatobiliary organogenesis, metabolic and secretory functions
[67]	In vitro-induced hepatic cells in LEM	iPSCs with ECs and MCs	Hepatocytes and cholangiocytes	Vascularized liver organoids were generated using induced hepatic tissue and dynamic liver-specific microenvironment as a drug testing platform
[64]	In vitro HBPO organoid structure	iPSCs with ECs and MSCs	Hepatocytes and cholangiocytes	Modeling of human hepato-biliary-pancreatic organogenesis from the foregut–midgut boundary
[68]	In vitro multi-cellular liver organoids composed of hepatocyte-, stellate-, and Kupffer-like cells	iPSCs and ECs	Hepatocyte-like cells, Kupffer-like cells, hepatic stellate-like cells	This model offers a new approach for studying inflammation and fibrosis in human liver disease such as steatohepatitis
[69]	In vitro chemically defined and serum-free environment	iPSCs and ESCs	Hepatocytes and cholangiocytes	Organized functional bile canaliculi system
[70]	In vitro expansion via modulation of Notch signaling	iPSCs	Hepatic endoderm, HscLCs, and endothelial cells	Functional vasculature and bile ducts in individual maps

Abbreviations: ASCs, adult stem cells; iPSCs, induced pluripotent stem cells; ESCs, embryonic stem cells; ECs, endothelial cells; MSCs, mesenchymal stem cells; OoC, organ-on-a-chip; (Lgr5+), stem cells expressing leucine-rich repeat-containing G-protein-coupled receptor 5; HscLCs, hepatic stellate cell-like cells; HBPO, hepatic–biliary–pancreatic organoid; mLO, multi-lineage liver organoids.

**Table 2:** The development of co-culture systems in liver organoid research.

Reference	Co-Culture System	Method	Results	Cell Source
[71]	Hollow fiber (HF) in bioreactor	HF membranes compartmentalize human hepatocytes on the external surface and between the fibers and compartmentalize endothelial cells into the fiber lumen	It retained its functional activity at high levels for up to 18 days	ASCs, ECs, hepatocytes
[72]	Spinning bioreactor	The spinning provides a flow suspension environment, enhances nutrient absorption, and promotes the self-assembly of cells into substantial functional	Generated self-assembled functional hepatobiliary organoids	ASCs (hepatocytes)
[67]	MOC	Microfluidic-based cell culture device with a continuous dynamic flow of media	The system produced functional vascularized liver organoids	iPSCs with ECs and MSCs
[73]	Multi-chamber chip	Multi-chamber chip for long-term co-culture of four tissue types	Multi-organoid chips consisting of functional intestine, liver, skin, and kidney organoids	iPSCs
[74]	Perfused tissues via synthetic 3D soft microfluidics	A 3D-printable 2-photon-polymerizable hydrogel formulation uniquely enables a 3D soft microfluidic strategy	The system enhanced tissue growth and differentiation compared to previously reported in vitro tissue vascularization strategies	iPSCs
[75]	In vitro MOC organoids on a microfluidic vascular bed	The microvascular bed consists of 64 microfluidic chips; each chip has a microfluidic chamber, which permits tissue grafting	The platform provided in vitro vascularization of tissues for routine grafting of spheroids, organoids, or (patient-derived) explants	ASCs (hepatocytes)

Abbreviations: HF, hollow fiber membranes; SB, spinning bioreactors; OoC, organ-on-a-chip; MOCs, multi-organ microfluidic chips; ASCs, adult stem cells; iPSCs, induced pluripotent stem cells; ECs, endothelial cells; MSCs, mesenchymal stem cells; (Lgr5+), stem cells expressing leucine-rich repeat-containing G-protein-coupled receptor 5.

**Table 3:** Summary and the progression of the application of organoids in liver diseases.

Rare Liver Disease	Co-Culture Type, Species	Major Findings	Reference
ALGS	- iPSC-derived hepatocyte	- The organoids had a regenerative property that is similar to that of the human liver, and a set of mutations related to ALGS was found to have a significant effect on the pathogenesis of liver disease.	[76]
Biliary Atresia	- Biliary atresia-BA-like model, human-derived	- Created BA model from a non-sick individual and demonstrated drug effectiveness.	[77]
		- The study found that beta-amyloid accumulates around bile ducts in patients’ livers.	[78]
		- Organoids derived from patients revealed molecular and functional evidence of delayed epithelial development in BA patients.	[79]
Wilson’s Disease	- Derived hepatocyte, dog model	- Survival of genetically corrected autologous organoid-derived hepatocyte-like cells in vivo.	[80]
		- Gene supplementation in hepatic organoids of COMMD1-deficient dogs restores function and can effectively cure copper storage disease.	[81]
Primary Sclerosing Cholangitis (PSC)	- Human bile duct organoid model	- Organoids recapitulate disease inflammatory immune profile.	[82]
		- Organoids recapitulate the senescence, pro-inflammatory factors, and macrophage recruitment observed in PSC.	[83]
Infectious Diseases			
HEV	- ASC human organoids inoculated with HEV particles in a transwell system	- This model can be used for drug screening, identifying new HEV inhibitors, and improving our insights to study virus–host interaction and antiviral therapies.	[84]
SARS-CoV-2	- ASC human liver bile duct organoids	- Liver damage caused directly by SARS-CoV-2 infection should be valued when treating COVID-19 patients.	[85]
Common Diseases with Liver Model			
ALF	- ASC mouse-derived model	- Human liver organoids generated with single-donor-derived multiple cells rescued mice from acute liver failure.	[86]
Steatohepatitis	- MOC human-derived organoid	- The model displayed genetic dysfunction of lysosomal acid lipase, which is found in severe steatohepatitis. The model can also be used to study inflammation and fibrosis in humans.	[68]
Ischemia	- Intrahepatic cholangiocyte organoids (ICOs), human-derived ASC	- The organoids recapitulate ischemic cholangiopathy in vitro and enable drug assessment studies for the discovery of new therapeutics for ischemic cholangiopathies.	[87]
NAFLD	- APOB or MTTP knockout organoids derived from human hepatocyte cell line	- This model facilitated steatosis etiology and provided a drug screening platform.	[88]
Diabetes Mellitus	- MOC model derived from humans	- This model showed the cellular functions of diabetic patients and their response to external stimuli and drugs.	[89]
Cystic Fibrosis	- ASC, human-derived, extrahepatic cholangiocyte organoids (ECOs)	- Organoid model derived from cystic fibrosis patient showed no CFTR channel activity but showed normal chloride channel and MDR1 transporter activity.	[52]

Abbreviations: ASCs, adult stem cells; iPSCs, induced pluripotent stem cells; ALGS, Alagille syndrome; BA, biliary atresia; PSCs, primary sclerosing cholangitis; SARS-CoV-2, severe acute respiratory syndrome coronavirus 2; ALF, acute liver failure; NAFLD, non-alcoholic fatty liver disease; MOC, multi-organ microfluidic chips; ICO, intrahepatic cholangiocyte organoids; ECO, extrahepatic cholangiocyte organoids.

## Data Availability

Not applicable.
